# *In Vitro* and *In Vivo* Antitumor Effect of Anti-CD33 Chimeric Receptor-Expressing EBV-CTL against CD33^+^ Acute Myeloid Leukemia

**DOI:** 10.1155/2012/683065

**Published:** 2012-01-05

**Authors:** A. Dutour, V. Marin, I. Pizzitola, S. Valsesia-Wittmann, D. Lee, E. Yvon, H. Finney, A. Lawson, M. Brenner, A. Biondi, E. Biagi, R. Rousseau

**Affiliations:** ^1^INSERM U590/Equipe Cytokines et Cancer, Centre Léon Bérard, 69373 Lyon Cedex 08, France; ^2^Centro Ricerca “M. Tettamanti”, Clinica Pediatrica Università Milano-Bicocca, Ospedale San Gerardo, Via Donizetti 106, 20052 Monza, Italy; ^3^Pediatrics, Cell Therapy Section, The University of Texas MD Anderson Cancer Center, Houston, 77030 TX, USA; ^4^Pediatric Hematology-Oncology, Center for Cell and Gene Therapy, Texas Children's Cancer Center, Baylor College of Medicine, Houston, 77030 TX, USA; ^5^UCB Celltech, 216 Bath Road, Slough, Berkshire SL1 3WE, UK; ^6^Pediatric Hematology-Oncology, Université Claude Bernard Lyon I, 69373 Lyon Cedex 08, France

## Abstract

Genetic engineering of T cells with chimeric T-cell receptors (CARs) is an attractive strategy to treat malignancies. It extends the range of antigens for adoptive T-cell immunotherapy, and major mechanisms of tumor escape are bypassed. With this strategy we redirected immune responses towards the CD33 antigen to target acute myeloid leukemia. To improve *in vivo* T-cell persistence, we modified human Epstein Barr Virus-(EBV-) specific cytotoxic T cells with an anti-CD33.CAR. Genetically modified T cells displayed EBV and HLA-unrestricted CD33 bispecificity *in vitro*. In addition, though showing a myeloablative activity, they did not irreversibly impair the clonogenic potential of normal CD34^+^ hematopoietic progenitors. Moreover, after intravenous administration into CD33^+^ human acute myeloid leukemia-bearing NOD-SCID mice, anti-CD33-EBV-specific T cells reached the tumor sites exerting antitumor activity *in vivo*. In conclusion, targeting CD33 by CAR-modified EBV-specific T cells may provide additional therapeutic benefit to AML patients as compared to conventional chemotherapy or transplantation regimens alone.

## 1. Introduction

Efforts to circumvent the limitations and improve the antitumor efficacy of current adoptive immunotherapy approaches have led to the development of novel strategies that combine the advantages of a T-cell-based therapy (tumor penetration, immune effector functions) and of antibody-based strategy (high specificity towards target antigen). The observation that engagement of a single T-cell receptor (TCRs) chain can induce cellular activation led to the design of chimeric antigen receptors (CAR), combining in a single molecule the antigen recognition (through motifs derived from single chain, highly variable antibody fragments, scFv) with signal transduction and activation motives (usually the TCR-*ζ* chain motif, CD3-*ζ*). CAR can be generated against every identified tumor-associated antigen (TAA) for which an antibody exists, including carbohydrates and glycolipids. The genetic modification of T cells to express CAR can redirect them toward tumor cells in a non-HLA-restricted manner. Nevertheless, the *in vivo* efficacy of CAR-expressing T cells is limited because the engagement of the CAR alone is usually not sufficient to mediate the critical costimulatory signals necessary for complete activation and persistence of genetically engineered T cells *in vivo* [1]. Expression of the CAR into antigen-specific cytotoxic T cells (CTLs) redirects the activated T cells (through their native TCR and costimulatory pathways) towards their new target [2–4]. The genetic modification of Epstein Barr Virus-specific-CTLs (EBV-CTLs) with tumor-specific CAR is particularly attractive because most individuals are persistently infected with EBV and express viral antigens in epithelial cells and B lymphocytes [5]. This approach has been validated in several recently published clinical trials [6–8].

CD33 is a myeloid-specific sialic acid-binding receptor overexpressed on the cell surface of 90% of acute myeloid leukemia (AML) blasts. CD33 has a role in regulating leukocyte functions in inflammatory and immune responses [9]. Normal granulomonocytic progenitors and mature cells express CD33, but not all normal hematopoietic stem cells [10, 11]. Conversely, AML stem cells express surface CD33 [12]. Gemtuzumab ozogamicin, a humanized anti-CD33 monoclonal antibody combined with calicheamicin (a cytostatic drug derived from anthracyclins), has demonstrated effective albeit short-lived antileukemic effects in clinical trials [13, 14]. Main side effects described in patients receiving this drug include myelosuppression, transient neutropenias, thrombocytopenias, and hepatic toxicity, which are related both to the expression of CD33 on myeloid progenitors and on Kuppfer cells and, most importantly, to the accumulation of free calicheamicin in the liver [13]. Contrary to other monoclonal antibody-based strategies, escape mechanisms towards gemtuzumab ozogamicin are not driven by downregulation of CD33 on tumor cells, but rather by chemoresistance due to the extracellular efflux of calicheamicin by ATP-dependant multidrug resistance (MDR) pumps [15, 16]. Thus, we hypothesized that an adoptive cellular therapy approach targeting CD33^+^ AML cells with CD33-specific CAR-expressing-EBV-CTLs should provide all the benefits of a T-cell-based immune effect: tumor cell killing in particular *via* an intrinsic antibody-dependant cellular cytotoxic effect, cytokine release, improved tumor penetration, and prolonged persistence compared to monoclonal antibody therapy. Furthermore, it should circumvent the chemoresistance mechanisms and the toxicity observed when combining a chemotherapeutic agent such as calicheamicin, with an anti-CD33 antibody, with the additional possibility to improve its safety profile, through the coexpression of a suicide gene [17].

We recently showed that the CD33-CAR approach effectively enhances the antileukemic activity of cytokine induced killer (CIK) cells [18], and, with this study, we extend our observation to EBV-CTLs, whose efficacy in the clinical setting has been widely documented, demonstrating that CD33-specific CAR-expressing EBV-CTLs can be redirected towards human CD33^+^ AML blasts *in vitro* and *in vivo* in a xenograft NOD-SCID mice model of AML.

## 2. Design and Methods

### 2.1. Cell Lines

The human CD33^+^ myeloid leukemia cell lines AML10 and ffLuc^+^ AML10 were generated and kindly provided by Dean Lee (The University of Texas MD Anderson Cancer Center, Houston, TX), after serial passages *in vivo *in NOD-SCID mice. AML10, ffLuc^+^ AML10, and KG-1 (human CD33^+^ AML, ATCC, Manassas, VA) cell lines were all maintained in RPMI (BioWhittaker, Walkersville, MD) supplemented with 10% heat-inactivated fetal calf serum (FCS, Hyclone, Logan, UT), 200 IU/mL penicillin, 200 *μ*g/mL streptomycin, and 2 mL glutamine (Invitrogen, Carlsbad, CA), further referred as complete medium. 293T cells (ATCCs) were cultured in IMDM (Invitrogen) supplemented as indicated above. Epstein-Barr virus B-lymphoblastoid cell lines (LCLs) were generated from six EBV-seropositive donors. For each donor, 5-10x10^6^ peripheral blood mononuclear cells (PBMC) were used for the establishment of EBV-LCLs by infection of PBMC with concentrated supernatants from the B95-8 working cell bank, as previously described [19].

### 2.2. Chimeric Receptor Cloning and Retrovirus Production

The high-affinity, humanized rat anti-human CD33, 113, in single-chain fragment variable-Fv-(L-(gly4ser)4-H generated using UCB's Selected Lymphocyte Antibody Method (kindly provided by Dr. Helene Finney, UCB Celltech, Slough, UK), was cloned in frame with CH2CH3-CD28 transmembrane-*ζ* domain in the SFG retroviral construct (kindly provided by Martin Pule, UCL, London, UK). Transient retroviral supernatant was produced by cotransfection of 293T cells with the MoMuLV *gag/pol* expression plasmid PeqPam3, the RD114 *env* expression plasmid, and the SFG-anti-CD33-*ζ* vector using GeneJuice transfection reagent (Calbiochem, San Diego, CA), according to manufacturers' instructions. The supernatant containing the retroviruses was harvested 2 and 3 days after transfection, snap-frozen, and stored at −80°C for further use.

### 2.3. Generation and Transduction of EBV-CTLs Lines

EBV-specific T-cell lines were generated as previously reported [20]. Briefly, PBMCs (2 × 10^6^ per well of a 24-well plate) were stimulated with autologous LCLs irradiated at 40 Gy at an effector-to-stimulator (E : S) ratio of 40 : 1. Starting on day 10, the responder cells were restimulated weekly with irradiated LCLs at an E : S ratio of 4 : 1, and rhIL-2 (50 U/mL) was added twice a week starting at the third stimulation. EBV-CTLs were transduced 48 to 72 hours after the third stimulation by irradiated autologous LCLs. CTLs were resuspended at 1 × 10^6^ cells/mL in complete culture medium and were incubated with 1.5 volume of retroviral supernatant for 48 hours at 37°C and 5% CO_2_. Transduced EBV-CTLs were further stimulated by weekly restimulations with irradiated autologous LCLs in the presence of rhIL-2 (50 U/mL).

### 2.4. Immunophenotyping

Gene-modified EBV-CTLs were analyzed for cell surface markers using flow cytometry. The following monoclonal antibodies were used: fluorescein isothiocyanate (FITC)—labeled mAb specific for human CD3, CD4, CD45RA; phycoerythrin (PE)—labeled mAb specific for human CD25, CD28, CD45RO, CD56, CD62L, CD69, CCR7, and TCR *γ*/*δ*; peridin chlorophyll protein (PerCP)—labeled mAb specific for the human CD8 (Becton Dickinson (BD) Biosciences (San Jose, CA)). Surface expression of the CD33-specific CAR was detected using a monoclonal antibody Fc-specific Cyanine-Cy5-conjugated (Fc-Cy5) (Jackson ImmunoResearch, West Grove, PA) recognizing the IgG1-CHCH3 component of the CAR. Cells were analyzed using a FACSCalibur flow cytometer (BD Biosciences). The human AML10 cells were analyzed for leukemic markers before and after contact with CD33-specific EBV-CTLs. The monoclonal antibodies used were FITC—labeled mAb specific for human CD33, CD38, CD123, CD13; PE—labeled mAb specific for human CD33, CD34. All gates were set using appropriate isotype control antibodies.

### 2.5. Expansion Rate Evaluation

After retroviral transduction, EBV-CTLs were rested in complete media without IL-2 for 2 days. Cells were then washed, counted, and plated at 1 × 10^6^ per well of a 24-well plate at a ratio of 4 : 1 with autologous LCLs. Cocultures were performed in the presence of low doses of rhIL-2 (20 U/mL). Expansion rate was assessed twice weekly by Trypan blue exclusion.

### 2.6. Determination of Specific Cytolysis by Chromium-Release Assay

The *in vitro* cytolytic activity of CAR-expressing EBV-CTLs was determined by ^51^Chromium-release assay. Briefly, EBV-CTLs were incubated for 4 hours with 5 × 10^3^ Na^51^CrO_4_ (^51^Cr, MP Biomedicals, Orangeburg, NY)—labeled target cells (KG-1, autologous and allogeneic LCLs). Effector cells (unmodified EBV-CTLs and anti-CD33.CAR-expressing EBV-CTLs) were harvested 7 and 28 days after transduction, washed, and incubated in triplicate with their ^51^Cr labeled targets. To determine whether cytolysis was restricted to MHC class I or CD33, target cells were incubated with anti-human HLA-A,B,C (clone W6/32) (Dako, Carpinteria, CA) or anti-CD33 (Chemicon, Temecula, CA, USA) monoclonal antibodies at 20 *μ*g/mL for 30 minutes at room temperature before the addition of effector cells. The percentage of specific cytolysis was calculated from the release of ^51^Cr, using a Cobra II AutoGAMMA (Canberra Packard Ltd, Pangbourne, Berks, UK). Data are reported as mean of ^51^Cr release ± standard deviation (SD).

### 2.7. Determination of IFN-*γ* and Granzyme B Release by Effector Cells Using the ELISpot Assay

Anti-CD33.CAR-expressing EBV-CTLs were cocultured with irradiated autologous LCLs and K562 cell line in an enzyme-linked immunospot (ELISpot) assay for IFN-*γ* and Granzyme B (GrB) (BD Biosciences), according to the manufacturer's instructions. The same effector cells (CAR-expressing EBV-CTLs) stimulated with 0.1 *μ*g/mL phorbol myristate acetate (PMA)/ionomycin (Sigma, St Louis, MI) were used as internal positive control. Effector cells (1 × 10^5^) were cultured in duplicate with 2 × 10^5^ of each specific target per well of a 24-well plate. Blocking experiments were performed using human anti-HLA I and anti-CD33 monoclonal antibodies, as described above, before the addition of effectors cells. Spots were developed according to the manufacturer's instructions and were enumerated using the Series 1 ImmunoSpot Image Analyzer and software (Cellular Technologies, Cleveland, OH).

### 2.8. Clonogenic Assay

Human bone marrow samples were obtained from commercial sources. Human CD34^+^ bone marrow cells were isolated using immunomagnetic beads (Miltenyi Biotech, Auburn, CA), incubated for 4 hours with unmodified or anti-CD33.CAR-expressing EBV-CTLs at an E : T ratio of 1 : 1 and plated at 2 × 10^4^ cells/dish in a methylcellulose-based medium (MethoCult H4434; StemCell Technologies Inc., Vancouver, BC, Canada). After 10 days, for each modified EBV-CTLs population, granulocyte monocytic colony-forming units (CFU-GMs), granulocyte, erythroid, macrophage, megakaryocyte colony-forming units (CFU-GEMMs), and erythroid burst-forming units (BFU-Es) colonies were counted. Results are represented as relative colony number compared with the control bone marrow progenitor CD34^+^ not exposed to any EBV-CTLs, set to 100%. To confirm the enumeration assays, colonies were collected and characterized by flow cytometry to analyze the expression of a set of surface markers specific for each colony type. The following antibodies were used: FITC—labeled anti human CD33 CD38, PE—labeled anti human CD38, CD54, CD71, and PerCP—labeled anti-human CD45RA, CD38, CD34 (BD Biosciences). Marked cells were analyzed using a FACSCalibur flow cytometer (BD Biosciences).

### 2.9. Xenograft Mouse Model and Treatment

On day 0, 6-week-old female NOD/SCID mice (Jackson Laboratory, Bar Harbor, ME) were subcutaneously injected with 2 × 10^6^ ffLuc^+^ AML10 cells. Beginning on day 5, tumor engraftment was evaluated *in vivo *using biophotonic imaging. Mice with growing tumors were divided into 4 treatment groups (8 mice/group) receiving weekly intravenous injections of saline serum, 5 × 10^6^ unmodified EBV-CTLs, or anti-CD33.CAR-expressing EBV-CTLs. Anesthetized mice were imaged using a Xenogen IVIS 100 series system (Xenogen, Alameda, CA) 15 minutes after intraperitoneal injection of 4.29 mg/mouse of D-luciferin potassium salt solution (Xenogen). Photons emitted from ffLuc^+^  AML10 xenografts were quantified using the Living Image software (Xenogen). Bioluminescence was measured as total photon flux normalized for exposure time and surface area and expressed in units of photons (p) per second per cm^2^ per steradian (sr) as previously validated [4]. Tumor response was monitored weekly over the treatment period. All animal experiments were performed according to institutional guidelines.

### 2.10. Histology and Immunofluorescence

Mice were euthanized 48 hours after the end of the treatment. Tumors were fixed in 10% formalin then embedded in paraffin. Sections (5 *μ*m) were used for routine hematoxylin/eosin histology and for immunofluorescence. Immunohistochemical detection of CD8^+^ EBV-CTLs and CD33 expression in the AML10 tumors was performed after blocking nonspecific binding with 10% normal goat serum in PBS for 30 minutes. Tumor sections were then washed in PBS and incubated with primary mAb (anti human CD8 or anti human CD33, Abcam, Cambridge, MA) at a 1 : 25 dilution for 1 hour at room temperature. Sections were then washed in PBS, incubated with the secondary antibody (FITC goat anti-mouse, Abcam) at a 1 : 100 dilution. After further washes, slides were counterstained with Hoechst solution (Sigma) during 10 minutes at 37°C, washed and mounted in Vectashield medium (Dako). Tissue sections were examined with a Zeiss Axiophot fluorescence microscope (Carl Zeiss Ltd., Hertfordshire, UK).

### 2.11. Statistical Analysis

All *in vitro *data are presented as mean ± standard deviation (SD). Student's *t*-test was used to determine statistical significance of differences between samples, and *P* value ≤ 0.05 was considered as indicating a significant difference. For the bioluminescent experiments, intensity signals were log-transformed and summarized using mean ± SD at baseline and multiple subsequent time points for each group of mice. Changes in intensity of signal from baseline at each time point were calculated and compared using paired *t* tests or Wilcoxon signed rank tests (Statview 5.0, SAS Institute Inc.) [4].

## 3. Results

### 3.1. Transduced EBV-CTLs Stably Express the Anti-CD33.CAR, While Maintaining Their Native EBV-CTLs Characteristics

**Figure 1:**
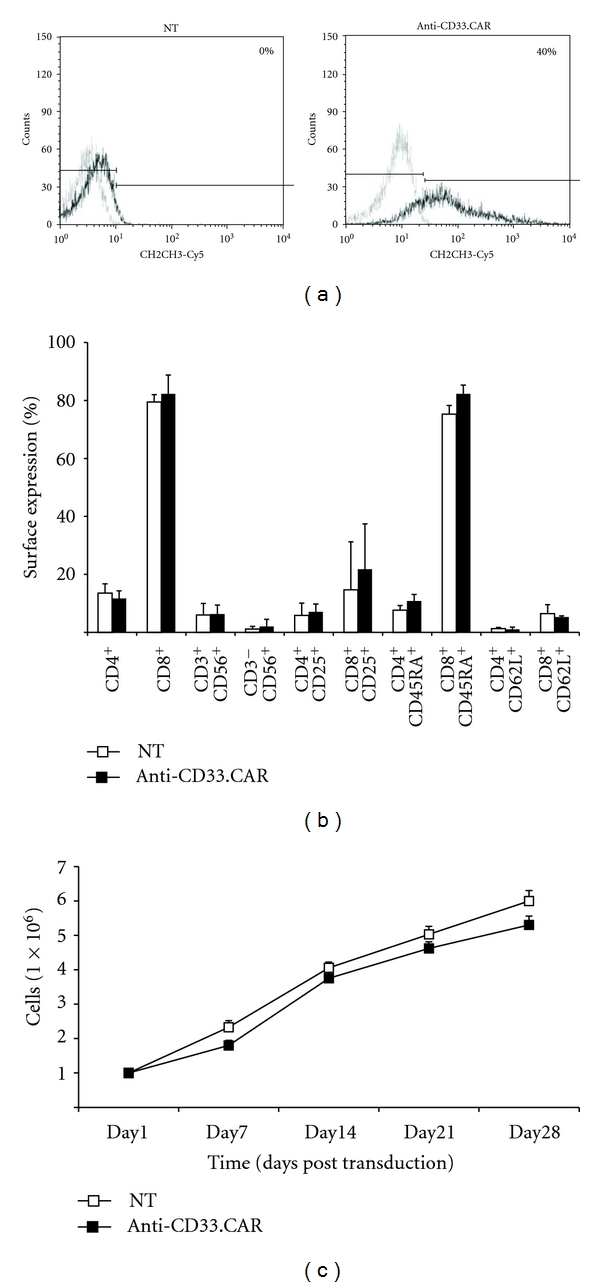
EBV-CTLs could be stably transduced with the anti-CD33.CAR without alteration in their native immunophenotype and expansion rate. (a) The expression of the anti-CD33.CAR on the surface of EBV-CTLs was evaluated by flow cytometry with a Cy5-conjugated-mAb specific for the CH2CH3 domain of the CAR after 7 days of culture. A representative plot of EBV-CTLs transduction after 7 days of culture is shown. (b) The expression of CD4, CD8, CD3 along with CD56, CD4, and CD8 along with CD25, CD4, and CD8 along with CD45RA, CD4, and CD8 along with CD62L on the surface of EBV-CTLs was evaluated after 30 days of culture by flow cytometry. (c) Proliferation of anti-CD33.CAR-transduced EBV-CTLs compared to unmanipulated EBV-CTLs was evaluated by cell count with Trypan blue exclusion after weekly stimulations at 4 : 1 ratio with either irradiated autologous LCLs. Cells were cultured with low-dose rhIL-2 (20 U/mL). Data shown are mean ± SD of 6 separate experiments.

EBV-CTLs from 6 EBV-seropositive donors were generated and retrovirally transduced to express the anti-CD33.CAR. The genetically modified EBV-CTLs were evaluated for expression of the CAR by flow cytometry using a mAb specific for the CH2CH3 domain. Genetically modified EBV-CTLs expressed the CAR (Figure 1(a)) 7 days after transduction, with a mean percentage of 35% ± 4% (*n* = 6) of positive cells. The transgene expression was still detected approximately at the same levels 1 month after transduction (mean percentage of CAR-expressing cells, 30% ± 5%). At this time point, the expression of CD4, CD8, CD3, CD56, CD25, CD45RA, and CD62L was unchanged in genetically modified EBV-CTLs compared to controls (Figure 1(b)). The expansion rate of anti-CD33.CAR-EBV-CTLs did not differ significantly upon coculture for 21 days with CD33^−^EBV^+^ autologous LCLs compared to unmodified EBV-CTLs (Figure 1(c)).

### 3.2. Anti-CD33.CAR-Transduced EBV-CTLs Are Functionally Bispecific

**Figure 2:**
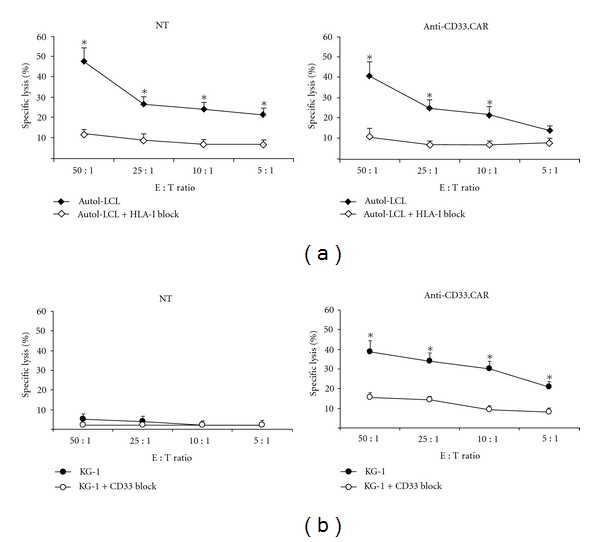
Anti-CD33.CAR-transduced EBV-CTLs efficiently killed autologous LCLs and CD33+ KG-1 cells. Cytotoxicity of unmanipulated EBV-CTLs and anti-CD33.CAR-transduced EBV-CTLs was evaluated by a standard 4-hour ^51^Chromium-release assay after 7 days of culture at effector:target (E : T) ratios of 50 : 1, 25 : 1, 10 : 1 and 5 : 1 against autologous LCLs (a) and CD33^+^ KG-1 cell line (b). To assess the specificity of either HLA-I-mediated and CAR-induced killing, target cells were preincubated with anti-human HLA-I (a) antibodies or an anti-human CD33 monoclonal antibody (b) before the addition of the effector cells. Data shown are mean ± SD of 6 separate experiments; *P ≤ 0.05.

We then investigated the ability of genetically modified CD33-specific EBV-CTLs to be activated and exert effector functions through their endogenous TCR and the transduced CD33-specific CAR. Four-hour chromium-release assays showed that the anti-CD33.CAR-transduced EBV-CTLs were able to kill autologous LCLs with the same efficiency as unmodified EBV-CTLs (mean lysis at 50 : 1 effector : target ratio of 40% ± 7%; *n* = 6, compared to a mean lysis of 48% ± 7%; *n* = 6 of untransduced EBV-CTLs) (Figure 2(a)). Incubation of autologous LCLs with MHC class I showed that MHC class I, as expected, was the major restriction element for the native antigen receptor function in both nontransduced and gene-modified EBV-CTLs, with a mean lysis at 50 : 1 effector : target ratio of 12% ± 2% and 11% ± 4%, respectively, for unmanipulated and anti-CD33.CAR-transduced EBV-CTLs (Figure 2(a), *P* ≤ 0.05). In addition anti-CD33.CAR-expressing EBV-CTLs could lyse efficiently the CD33^+^ expressing KG-1 cell line (mean lysis at 50 : 1 effector : target ratio of 39% ± 6%; *n* = 6 compared to a mean lysis of 5% ± 3% of untransduced EBV-CTLs; *n* = 6; *P* ≤ 0.05). Preincubation of CD33^+^ KG-1 target cells with a CD33-blocking MoAb resulted in a significant inhibition of lysis (up to 40%) by CD33-specific EBV-CTLs, with a mean lysis at 50 : 1 effector : target ratio of 15% ± 2% (Figure 2(b)).

**Figure 3:**
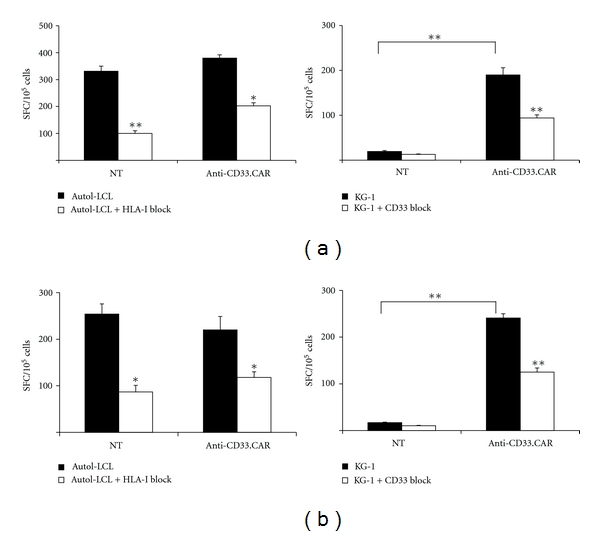
Anti-CD33.CAR-transduced EBV-CTLs released substantial levels of IFN-*γ* and Granzyme B when stimulated with either autologous LCLs and CD33^+^ KG-1 cells. Release of IFN-*γ* (a) and Granzyme B (b) from unmanipulated or anti-CD33.CAR-transduced EBV-CTLs was evaluated by ELISpot assays after stimulation with either irradiated autologous LCLs or irradiated KG-1 cells at 1 : 2 ratio. To assess the specificity of either HLA-I-mediated and CAR-induced cytokine release target cells were preincubated with anti-human HLA-I antibodies or an anti-human CD33 monoclonal antibody before the addition of the effector cells. Data shown are mean ± SD of 6 separate experiments; *P ≤ 0.05; **P ≤ 0.005.

The ability of CD33-specific EBV-CTLs to secrete cytokines in response to both EBV^+^ and CD33^+^ targets was investigated by ELISpot assay after culturing target cells either with unmodified or genetically-modified EBV-CTLs. Levels of Th1 (IFN-*γ*) and Tc (GrB) cytokines released by modified and unmodified EBV-CTLs in response to EBV^+^ targets were almost similar (mean spot forming cells-SFC-/10^5^ cells of 380 ± 12 and  331 ± 19, respectively, for anti-CD33.CAR-transduced EBV-CTLs and unmanipulated EBV-CTLs for IFN-*γ*, (Figure 3(a), left panel) and mean SFC/10^5^ cells of 220 ± 29 and  254 ± 22, respectively, for anti-CD33.CAR and unmanipulated EBV-CTLs for GrB, (Figure 3(b), left panel). This response to EBV^+^ cells was mediated by the native TCR as demonstrated by blocking class I MHC antigens on target cells which significantly reduced the cytokine release by 40 to 50% (Figures 3(a) and 3(b), left panel). No production of cytokines was detectable after exposure of unmodified EBV-CTLs to CD33^+^ targets, whereas IFN-*γ* and GrB cytokine production was seen in response to stimulation of anti-CD33.CAR-transduced EBV-CTLs by CD33^+^ cells (mean SFC/10^5^ cells of  190 ± 16  for IFN-*γ* and mean SFC/10^5^ cells of  241 ± 9  for GrB) (Figures 3(a) and 3(b), right panel). This activity was mediated by the chimeric receptor pathway since CD33 MoAb significantly blocked IFN-*γ* and GrB secretion in a range of 40 to 50% (Figures 3(a) and 3(b), right panel).

### 3.3. Anti-CD33.CAR-Transduced EBV-CTLs Have a Myeloablative Activity *In Vitro*, But the Colony-Forming Capacity Is Maintained

**Figure 4:**
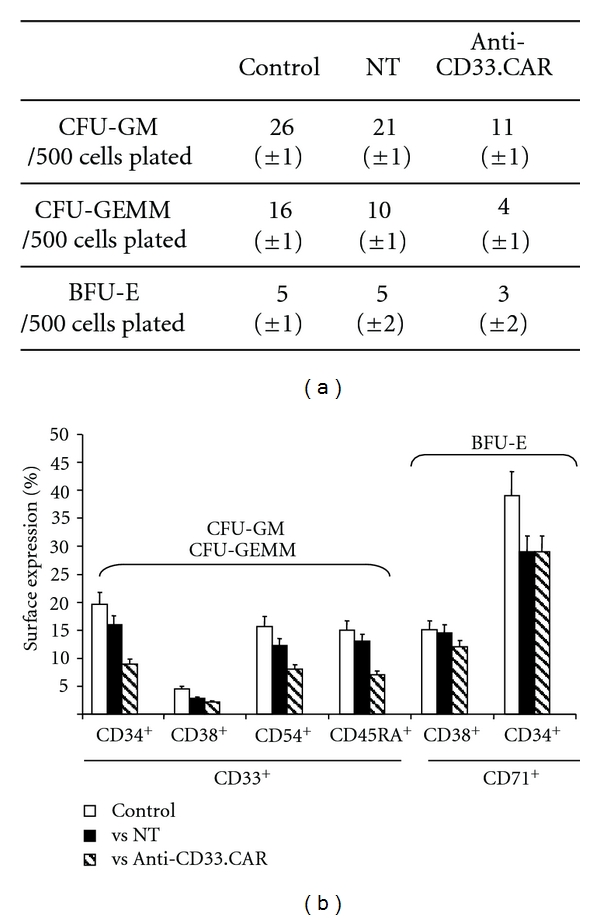
Cytotoxicity of anti-CD33.CAR-transduced EBV-CTLs against normal myeloid progenitors. (a) Unmanipulated or anti-CD33.CAR-transduced EBV-CTLs were incubated with bone-marrow-derived CD34^+^ progenitors at E : T ratio of 1 : 1 for 4 hours. Cells were then seeded in methylcellulose-based medium and after 10 days CFU-GM, CFU-GEMM, and BFU-E were counted. Data shown are mean ± SD of 6 independent experiments. (b) Colonies were collected and characterized by flow cytometry to assess the expression of surface markers specific for each colony type. Data shown are mean ± SD of 6 independent experiments.

Exposure of CD34^+^ bone marrow progenitor cells to unmodified EBV-CTLs was associated with a mean reduction in colony number of 20% for CFU-GM and 38% for CFU-GEMM compared to the control (Figure 4(a)), whereas anti-CD33.CAR-EBV-CTLs significantly reduced the CFU counts by 58% to 75% compared to the control (*P* ≤ 0.005) (Figure 4(a)). BFU-E subpopulation, which does not express CD33, was not affected by the activity of anti-CD33.CAR-transduced EBV-CTLs. These results were confirmed by flow cytometry analysis, which showed a significant decrease (*P* ≤ 0.05) in the expression of cell surface markers of CFU-GEMM and CFU-GM, with BFU-E being not affected (Figure 4(b)). Overall, our results indicate that despite anti-CD33.CAR-transduced EBV-CTLs had a cytolytic action on CD34^+^ progenitor cells, those remained capable to generate normal progeny.

### 3.4. Anti-AML Effect of Anti-CD33.CAR-Transduced EBV-CTLs *In Vivo*

**Figure 5:**
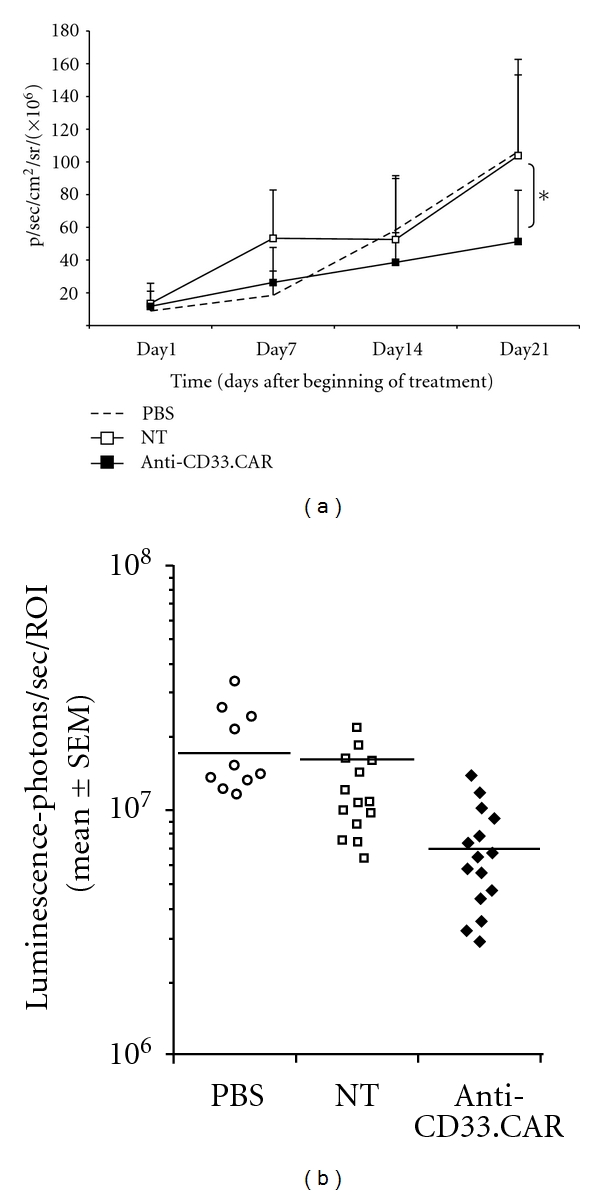
*In vivo* activity of anti-CD33.CAR-transduced EBV-CTLs against AML10. NOD/SCID mice were subcutaneously injected with ffLUC+AML10 cells, and after 5 days mice were weekly intravenously injected with either PBS, unmanipulated or anti-CD33.CAR-transduced EBV-CTLs. (a) Tumor volume was monitored weekly after D-luciferin injection using a Living Image software and was quantified as total photon flux normalized for exposure time and surface area and expressed in units of photons (p) per second per cm^2^ per steradian (sr). Data shown are mean ± SD of 8 mice/group; *P ≤ 0.05. (b) Intensity signals were log-transformed and summarized using mean ± SD at baseline and multiple subsequent time points for each group of mice. Changes in intensity of signal from baseline at each time point were calculated.

When we analyzed the *in vivo* antitumor activity of anti-CD33.CAR genetically modified EBV-CTLs in AML10-bearing mice, we observed that, compared to untreated mice or to mice receiving EBV-CTLs, anti-CD33.CAR-expressing EBV-CTLs reduced tumor progression (tumor inhibition rate 43% *versus *12%, *P* ≤ 0.05, Wilcoxon rank sum test) (Figures 5(a) and 5(b)).

**Figure 6:**
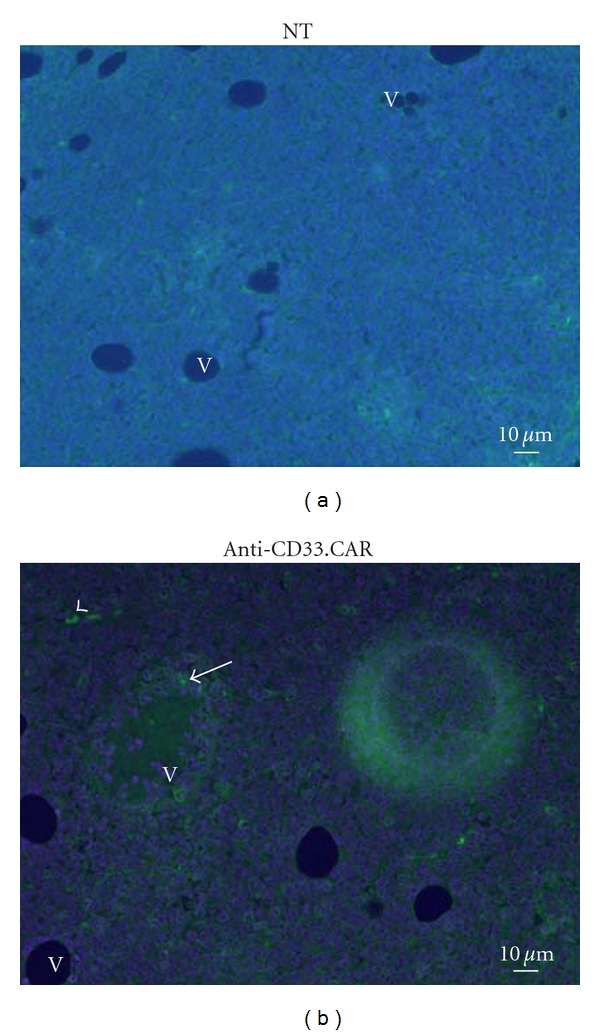
Infiltration of tumors by EBV-CTLs. Forty-eight hours after i.v. administration of EBV-CTLs, tumors were resected, fixed in 10% formalin solution, and embedded in paraffin. CD8^+^ EBV-CTLs were detected by immunohistochemistry using an anti-CD8 antibody and reported in (a) unmodified EBV-CTLs, (b) CD33-EBV-CTLs. Genetically modified T cells were found at the periphery of the tumor in the vicinity of vessels (indicated as “V”), isolated or forming cluster (arrowheads or arrows, resp.).

Immunohistochemistry performed on tumors analyzed 48 hours after i.v. administration of EBV-CTLs seems to indicate that anti-CD33.CAR-EBV-CTLs were able to infiltrate the tumor (Figure 6). Genetically modified T cells were found at the periphery of the tumor in the vicinity of vessels, isolated or forming cluster (arrowheads or arrows, resp.).

## 4. Discussion

Our results show that the expression of CD33 chimeric receptor in EBV-specific CTLs, while does not affect their phenotype and does not interfere with their ability to proliferate or to respond to autologous EBV-infected targets, renders them capable to specifically lyse CD33^+^ target cells and release Th1 and Tc cytokines upon encounter with target cells, even though the low average transduction efficiency. In order to further improve this percentage and possibly augmenting CAR^+^ T-cell response to its specific target, we might concentrate anti-CD33.CAR-specific retroviral supernatant administration by increasing the Multiplicity of Infection (MOI)/cells ratio. However, more efforts will be needed to find new regulatory elements such as insulators, tissue-specific promoters, or ribosome-binding sites, alone or in combination, to be included in the specific transgene plasmid in order to increase transcription efficiency in T cells. This will certainly be accompanied by a decreased of the *in vitro* E : T ratio, increasing the killing capacity of the transduced T cells, phenomenon that we can predict would also have an impact in the T-cell efficiency after clinical infusion. These results confirm that the engagement of the chimeric receptor by tumor antigen initiates signalling to the T-cell nucleus and induces effector functions, including cytokine secretion and specific lysis of antigen-expressing tumor cells *in vitro*. T-cell retargeting operates in an MHC unrestricted manner to attack the tumor, whereas it retains MHC-restricted specificity for the endogenous TCR. When we analyzed the *in vivo* activity of anti-CD33.CAR genetically modified EBV-CTLs in a mouse model of AML, we observed that intravenous administration of CD33-redirected EBV-CTLs in AML-bearing mice can exert a significant albeit partial antileukemic activity. The incomplete antitumoral effect exerted by anti-CD33.CAR-transduced EBV-CTLs might be related to their limited persistence *in vivo* in this animal model, an expected outcome given that CTLs were injected intravenously without concomitant administration of recombinant human IL-2 and the lack of chronic EBV stimulation in NOD/SCID mice. Indeed, studies reporting complete regression of tumor in mice were performed by intratumoral injection of T cells expressing chimeric receptors and/or addition of intravenous IL-2, or vaccination with autologous LCLs [3, 21, 22], which may have allowed for improved T-cell survival and *in vivo* expansion. Of note, despite the lack of cytokine addition or EBV stimulation, anti-CD33.CAR-transduced EBV-CTLs were able to home and infiltrate the tumor, where they exerted a significant anti-tumor activity. In addition, we observed that the repeated administrations of anti-CD33.CAR-transduced EBV-CTLs did not result in the selection of CD33-negative tumor clones, that would be resistant to our therapeutic approach.

Even though results form a recent phase I clinical trial with EBV-CTLs genetically modified with a chimeric receptor specific for the GD2 antigen carrying just the *ζ* intracellular domain clearly indicate that these cells are very effective in terms of anti-tumor activity and that their efficacy is sustained by their capacity to persist upon infusion [8], other strategies can be exploited to improve their persistence. One possibility is represented by coexpression of genes coding for homeostatic cytokines, such as IL-15 [23], or the vaccination with LCLs, to promote the physiologic restimulation of EBV-CTLs trough their native TCR, or lymphodepletion prior to the EBV-CTLs infusion, to remove T-suppressor cells and recreate a more favourable cytokines milieu [24, 25]. In addition, the current availability of new immunodeficient models (e.g., NOD/SCID/*γ*c^−/−^) [26, 27] might help in better characterizing the *in vivo* anti-tumor activity of these cells in preclinical settings. Indeed, when moving to the clinical setting, this kind of cell therapy approach could be further improved by various strategies, as already exploited for other types of tumor: patients could receive preventively a short schedule of chemotherapy to decrease the burden of disease, and, immediately before the injection, a lymphodepletive fludarabin-based scheme could be also added to favour the expansion of cells after infusion and the depletion of regulatory T cells. Moreover, addition of systemic administration of clinical-grade IL-2, IL-15, or IL-7 could be of further help to improve cell expansion and survival.

Taken together our data highlight that CD33^+^AML adoptive immunotherapy using CD33-specific EBV-CTLs might be an alternative strategy to an antibody-based therapy such as gemtuzumab ozogamicin. This is particularly relevant in light of the frequently and severe reported hepatic toxicity related to the addition of a toxic compound to the CD33 targeting and subsequent calicheamicin accumulation and/or unwanted release and toxicity to normal cells surrounding CD33^+^ tumor cells. Another advantage of CD33-specific EBV-CTLs over gemtuzumab ozogamicin is their independence from the MDR1 pathway, a known factor of chemoresistance to calicheamicin. Moreover, in immunocompromised patients, the administration of chimeric T cells endowed *per se* with anti-tumoral activity may prove more effective than the use of monoclonal antibodies, whose efficacy is impaired by dysfunctional or absent immune effectors. Finally, in patients with CD33^+^ AML, leukemic stem cells are considered a major cause of relapse based on their repopulating capacity and thus represent a critical target for AML-specific therapies [28, 29]. Since AML stem cells reside within the CD34^+^CD38^−^CD33^+^ compartment of the leukemic clone [12, 30], eradication of this subpopulation by targeting CD33 is predicted to give therapeutic benefit to treated patients.

On the other side, concerning the potential toxicity on the normal myeloid compartment, our results indicate that, at least *in vitro*, anti-CD33.CAR-transduced-EBV-CTLs did not irreversibly impair the functions of CD33^+^ hematopoietic progenitors, as confirmed in a recently published paper using CIK cells expressing anti-CD33.CAR [18]. This might be partially explained by the fact that not all normal human hematopoietic progenitors express CD33 on their surface [12]. We are now in the process of corroborating these data in a murine NOD/SCID/*γ*c^−/−^ model reconstituted with normal hematopoietic progenitors. Anyhow, we are well aware of the potential risks in humans, and, certainly, first trials will necessarily require a dose-escalation scheme, starting form very low doses and progressively increasing towards higher numbers of infused cells. Moreover, a construct containing a suicide gene would be highly desirable (as discussed at the end), so that, in case of unwanted or severe toxicity, cells can be rapidly killed. More selective antigens for the AML leukaemia stem cell are under investigation (such as CD123 or CD44 [28, 29]), in case of limiting myeloid toxicity related to the CD33 targeting.

Following ongoing clinical applications of chimeric T cells in B-cell malignancies [31, 32], the development of CD33-targeting approaches using chimeric EBV-specific T cells includes the generation of anti-CD33 chimeric EBV-CTLs from the stem cell donor and the subsequent administration to patients with early relapse following allogeneic bone marrow transplantation, either preemptively or prophylactically. In such a circumstance, since the toxicity towards the normal myeloid compartment is still unknown, an approach to increase the safety of modified anti-CD33.CAR cells would be desirable, such as the addition of a suicide gene, as described by our group elsewhere [18].
